# Endothelial cell‐specific reduction in mTOR ameliorates age‐related arterial and metabolic dysfunction

**DOI:** 10.1111/acel.14040

**Published:** 2023-11-28

**Authors:** Md Torikul Islam, Shelby A. Hall, Tavia Dutson, Samuel I. Bloom, R. Colton Bramwell, John Kim, Jordan R. Tucker, Daniel R. Machin, Anthony J. Donato, Lisa A. Lesniewski

**Affiliations:** ^1^ Department of Nutrition and Integrative Physiology The University of Utah Salt Lake City Utah USA; ^2^ Division of Geriatrics, Department of Internal Medicine The University of Utah School of Medicine Salt Lake City Utah USA; ^3^ Nora Eccles Harrison Cardiovascular Research and Training Institute The University of Utah Salt Lake City Utah USA; ^4^ Geriatric Research Education and Clinical Center Veteran's Affairs Medical Center Salt Lake City Utah USA; ^5^ Department of Biochemistry The University of Utah Salt Lake City Utah USA

**Keywords:** aging, arterial stiffness, endothelial cells, inflammation, metabolic function, oxidative stress, senescence, vasodilation

## Abstract

Systemic inhibition of the mammalian target of rapamycin (mTOR) delays aging and many age‐related conditions including arterial and metabolic dysfunction. However, the mechanisms and tissues involved in these beneficial effects remain largely unknown. Here, we demonstrate that activation of S6K, a downstream target of mTOR, is increased in arteries with advancing age, and that this occurs preferentially in the endothelium compared with the vascular smooth muscle. Induced endothelial cell‐specific deletion of mTOR reduced protein expression by 60–70%. Although this did not significantly alter arterial and metabolic function in young mice, endothelial mTOR reduction reversed arterial stiffening and improved endothelium‐dependent dilation (EDD) in old mice, indicating an improvement in age‐related arterial dysfunction. Improvement in arterial function in old mice was concomitant with reductions in arterial cellular senescence, inflammation, and oxidative stress. The reduction in endothelial mTOR also improved glucose tolerance in old mice, and this was associated with attenuated hepatic gluconeogenesis and improved lipid tolerance, but was independent of alterations in peripheral insulin sensitivity, pancreatic beta cell function, or fasted plasma lipids in old mice. Lastly, we found that endothelial mTOR reduction suppressed gene expression of senescence and inflammatory markers in endothelial‐rich (i.e., lung) and metabolically active organs (i.e., liver and adipose tissue), which may have contributed to the improvement in metabolic function in old mice. This is the first evidence demonstrating that reducing endothelial mTOR in old age improves arterial and metabolic function. These findings have implications for future drug development.

## Graphical abstract

Although systemic mTOR inhibition improves age‐related physiological dysfunction, the cell types that mediate the beneficial effects remain elusive. Here, we demonstrate that endothelial cell‐specific reduction of mTOR ameliorates arterial and metabolic function in old age. These improvements are concomitant with reduced senescence burden, inflammation, and oxidative stress in arteries, lung, adipose tissue, and liver. These results support the exploration of therapeutic strategies targeting endothelial mTOR signaling.

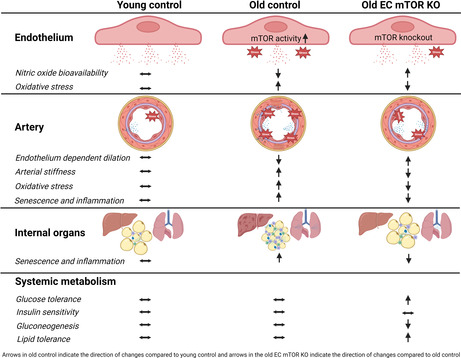

**Abbreviations**
ACh: acetylcholine
CVD: cardiovascular disease
EC: endothelial cell
EDD: endothelium‐dependent dilation
EPR: electron paramagnetic resonance
GSIS: glucose‐stimulated insulin secretion
GTT: glucose tolerance test
ITT: insulin tolerance test
KO: knockout
LTT: lipid tolerance test
mTOR: mammalian target of rapamycin
NO: nitric oxide
pgWAT: perigonadal white adipose tissue
PTT: pyruvate tolerance test
PWV: pulse wave velocity
VSMC: vascular smooth muscle cell
WT: wildtype

## 1 INTRODUCTION

Cardiovascular diseases (CVDs) and metabolic diseases are leading causes of global mortality and morbidity in older adults (Donato et al., 2018; Islam et al., 2020). Deciphering the mechanisms and identifying novel therapeutic targets is imperative to address this public health burden. Although advancing age adversely affects a variety of organ systems, endothelial cell dysfunction is one of the earliest events that is linked to CVD and metabolic dysfunction (Donato et al., 2018; Graupera & Claret, 2018). The endothelium is a monolayer of cells that resides on the inner lumen of the vasculature and separates two key body compartments—circulating blood and peripheral organs (Bloom et al., 2022; Islam, Cai, et al., 2023). Endothelial cells play critical roles in the development and maintenance of the vascular network and thus nutrient and oxygen delivery in all organs and tissues (Bloom et al., 2022). Therefore, dysfunction in endothelial cells may result in detrimental consequences across many organs and tissues (Bloom et al., 2022; Donato et al., 2018). Age‐related endothelial dysfunction is characterized by elevated markers of cellular senescence, inflammation, and oxidative stress (Bloom et al., 2023; Islam et al., 2021). Inflammation and oxidative stress attenuate the bioavailability of nitric oxide (NO), a key endothelial‐derived vasodilatory molecule, resulting in impairments in endothelial‐dependent dilation (EDD) (Donato et al., 2018; Islam et al., 2021). Furthermore, endothelial‐derived inflammatory cytokines, chemokines, extracellular matrix proteins, and reactive oxygen species modulate vascular smooth cell function contributing to arterial stiffness (Donato et al., 2018; Martens & Seals, 2016). Together, impaired EDD and elevated arterial stiffness are the major components of arterial dysfunction that predict and lead to clinical CVDs (Donato et al., 2018; Islam et al., 2021; Martens & Seals, 2016). Emerging evidence also suggests that the vascular endothelium is a critical regulator of systemic metabolic function (Graupera & Claret, 2018; Pi et al., 2018). Indeed, endothelial cell dysfunction is sufficient to induce systemic metabolic dysfunction (Graupera & Claret, 2018; Hasegawa et al., 2012; Yokoyama et al., 2014). Therefore, elucidating the underlying mechanisms of age‐related endothelial dysfunction, its vascular and metabolic consequences, and identifying endothelial‐specific therapeutic targets may offer multiple benefits in older adults.

The mammalian target of rapamycin (mTOR) is a serine/threonine kinase that regulates diverse cellular and physiological processes spanning across development, growth, and pathologies (Liu & Sabatini, 2020). The mTOR signaling pathway works via two distinct protein complexes known as mTOR complex 1 (mTORC1) and mTOR complex 2 (mTORC2) (Liu & Sabatini, 2020). While mTORC1 primarily regulates protein translation as well as nucleotide and lipid biosynthesis, mTORC2 is involved in cell survival and proliferation (Liu & Sabatini, 2020). The mTOR signaling pathway is of great interest across many fields, in part due to the ability of rapamycin, a pharmacological mTOR inhibitor, to extend lifespan in various model organisms suggesting that the mTOR signaling pathways can be a target for age‐related physiological dysfunction (Harrison et al., 2009; Lamming et al., 2013). To date, systemic mTOR inhibition has been demonstrated to ameliorate a host of age‐related phenotypes, including arterial stiffening, impaired EDD, and metabolic dysfunction (Donato et al., 2018; Johnson et al., 2013; Liu & Sabatini, 2020). However, the tissues or organs that mediate the beneficial effects of systemic mTOR inhibition in aging and age‐related diseases remain largely unknown.

Although systemic mTOR inhibition in old age confers many beneficial effects, in most organs, the downstream effectors of mTOR (i.e., p‐S6K) do not increase with aging (Baar et al., 2016). However, we and others have shown that with advancing age p‐S6K increases in arteries (Baar et al., 2016; Lesniewski et al., 2017). Moreover, it has been demonstrated that higher p‐S6K expression is associated with cellular senescence, inflammation, and oxidative stress in human umbilical vein endothelial cells, which was suppressed by mTOR inhibition (Rajapakse et al., 2011; Yang et al., 2018). In this present study, we tested the hypothesis that endothelial cell mTOR activity increases with aging and that a reduction in endothelial‐specific mTOR will ameliorate age‐related arterial and metabolic dysfunction. We also hypothesized that these improvements will be associated with a reduction in senescence, inflammation, and oxidative stress. To do so, we first examined the p‐S6K expression in aortic endothelial and vascular smooth muscle cells from young and old C57BL/6 mice. We next generated a tamoxifen‐inducible endothelial cell‐specific mTOR knockout (KO) mouse model. To examine arterial function, we assessed arterial stiffness and EDD in young and old KO and wild‐type (WT) mice. We also measured expression of senescence, inflammation, and oxidative stress markers as well as assessed the contribution of reactive oxygen species to the improvement of EDD in old mice. To examine metabolic function, we performed a battery of metabolic assessments including glucose‐, insulin‐, pyruvate‐, and lipid‐tolerance tests as well as a glucose‐stimulated insulin secretion assay. Lastly, we examined the impact of endothelial mTOR reduction on markers of senescence and inflammation in endothelial‐rich and/or metabolically active tissues.

## 2 METHODS AND MATERIALS

### 2.1 Ethical approval

All animal procedures conform to the Guide to the Care and Use of Laboratory Animals: Eighth Edition (Council 2010) and were approved by the University of Utah and Veteran's Affairs Medical Center‐Salt Lake City Animal Care and Use Committees.

### 2.2 Animals

Young (4–6 month) and old (22–24 month) C57BL/6J mice were used for assessing age‐related changes in endothelial cell and vascular smooth muscle cell mTOR activation. Young mice were purchased from Charles River, and old mice were obtained from the National Institute on Aging rodent colony maintained at Charles River Inc. We generated a tamoxifen‐inducible endothelial cell‐specific mTOR KO mouse model by crossing mTOR floxed (mTOR^f/f^) with cadherin 5 Cre transgenic (Cdh5 cre+) mice (Figure S1A) obtained from our collaborators Dr. Matthew Rondina and Dr. Dean Li, respectively. Both of these transgenic mouse strains were generated on a C57BL/6J background. The Chd5 Cre + strain used was originally developed and described by Dr. Ralf H. Adams (Sörensen et al., 2009). The mTOR^f/f^ mice were created by Dr. Sara C. Kozma (Gangloff et al., 2004). To identify the KO and WT littermates, we genotyped the pups using polymerase chain reaction and agarose gel technique (Figure S1B). Primer sequences for genotyping are provided in Table S2. Mice were born according to the normal Mendelian ratio. We studied young (4–6 month) and old (22–24 month) mice. Both male and female mice were included in this study. Deletion of mTOR was induced by 4 consecutive doses of tamoxifen (4mg/day, oral gavage). We performed functional experiments two months after the induction of endothelial mTOR deletion. During this period, we administered a weekly maintenance dose of tamoxifen (4mg, oral gavage). We analyzed male and female mice separately for the key outcomes and we did not observe any sex‐specific effects on metabolic and arterial function in old WT and KO mice (Figure S2). Therefore, in the main figures, we pooled data from male and female mice together. All mice were maintained in the Salt Lake City VA Medical Center's Animal Facility in standard shoe box cages on a 12:12 light: dark cycle with water and food ad libitum.

### 2.3 Aorta preparation, staining, and imaging

For aortic preparation, mice were sacrificed by exsanguination via cardiac puncture while under isoflurane anesthesia, a bilateral thoracotomy was performed to ensure euthanasia. The thoracic cavity was opened, and the portal vein was cut using scissors. Mice were perfused with saline using an 18G needle through left ventricle of the heart. Saline perfusion was continued until a clear saline was seen to come out via portal vein, indicating that the blood was flushed from the vascular system. After saline perfusion, mice were perfusion fixed with 50 mL of 4% paraformaldehyde (pH 7.4) for a duration of ~5 min. Stiffness of the carcass was used as an indicator of good paraformaldehyde fixation. The aorta was dissected and cleaned of perivascular tissues under a dissection microscope. Subsequent procedures were performed in 0.2 mL PCR tubes, and the aortic samples were transferred between tubes containing the required solutions.

The samples were dehydrated in 100% methanol for 15 min at −20°C followed by rehydration in phosphate‐buffered saline (PBS) 3 × 5 min. The samples were incubated in a blocking solution (1 mg/mL bovine serum albumin, 3% goat serum, 0.1% triton X‐100, 1 mM EDTA in PBS) for 30 min at room temperature. Samples were then incubated in the primary antibodies against p‐S6K (1:100; Thermo Fisher; p70 (S6K) Rabbit pAb #14485‐1‐AP) and mTOR (1:200; Cell Signaling; mTOR Rabbit mAb #2983) for 1 h at room temperature. Following incubation with primary antibodies, samples were washed in PBS (3 × 5 min), followed by incubation with an Alexa Fluor 555 secondary antibody (1:500; AF 555; Invitrogen) for 1 h at room temperature. Samples were then washed again in PBS (3 × 5 min) and then cut open longitudinally, placed on a glass coverslip containing a drop of DAPI Fluoromount‐G (VWR; #102092‐102) with the inner lumen facing down. A glass slide was placed onto the outer side of the aorta and the samples were weighed by ~1 kg weight for 5 min and stored in a dark place until imaging.

Z‐stack images were captured using an Olympus Fluoview FV1000 Confocal microscope. Endothelial cells and vascular smooth muscle cells were distinguished by distinct nuclear morphology with DAPI and basement membrane. Therefore, despite a lack of cell‐type specific protein markers, we are confident in identifying endothelial cells and vascular smooth muscle cells and quantifying the fluorescence intensity of the proteins of interest. Fluorescence intensity of the proteins from the Z‐stack images was quantified using Fiji ImageJ software according to the established protocol in our laboratory (available from authors upon request). Since all the staining was performed together using the same conditions and images were captured using the same confocal settings, we did not use a house‐keeping protein to normalize the signal intensity. Likewise, anti‐mouse immunofluorescence antibodies against total mTOR and total S6K that are made in species other than the species for p‐mTOR and p‐S6K were not available at the time when we performed this study, hindering our ability to normalize p‐mTOR and p‐S6K to total mTOR and total S6K, respectively.

### 2.4 Isolation of endothelial cells

Mouse lungs were collected, minced using sterile scissors, digested by collagenase‐I (Worthington Biochemical Co/ LS004194), and filtered through a 70 μm strainer. Cell suspensions were incubated with PECAM‐conjugated Dynabeads. Cells were seeded in 100 mm cell culture dishes at 37°C in an incubator. When the cells were confluent, the cell suspensions were sorted using ICAM‐conjugated Dynabeads. The sorted cells were plated into 6‐well plate cell culture dishes. When cells were 70–80% confluent, they were treated with culture media containing 1 μM hydroxy tamoxifen for 24 h followed by another 24 h recovery period in untreated culture media. At the end of treatment and recovery, cells were collected for protein extraction and western blotting.

### 2.5 Western blotting

Protein lysates were prepared from primary lung endothelial cells using ice‐cold RIPA buffer (Sigma Aldrich) containing proteases and phosphatase inhibitor cocktails (Thermo Fisher) as described previously (Gogulamudi et al., 2023). Protein concentration was measured using BCA assay (Thermo Fisher) according to the manufacturer's protocol. Protein expression was measured by standard western blot procedures using anti‐mouse primary antibodies against total mTOR (mTOR; 1:1000; 289 kDa; Cell Signaling) and vinculin (hVIN‐1, monoclonal; 1:1000; 116 kDa, Sigma Aldrich). Goat Anti‐Rabbit IgG (H + L)‐HRP Conjugate (Bio‐Rad) and Goat Anti‐Mouse IgG (H + L)‐HRP Conjugate (Bio‐Rad) were used as the secondary antibody. Images were visualized and quantified using Bio‐Rad ChemiDoc™ XRS+ with Image Lab™ Software.

### 2.6 Aortic stiffness

Aortic stiffness was assessed by measuring aortic pulse wave velocity (PWV), as described previously (Machin et al., 2020, 2023). Briefly, mice were anesthetized with 2% isoflurane and 98% oxygen at 2 L/min flow rate and placed in the supine position on a heated platform (37°C). Pulse waveforms at the transverse aortic arch and at the abdominal aorta were measured simultaneously with 20‐MHz Doppler probes (Indus Instruments) and recorded using WinDAQ Pro + software (DataQ Instruments). After waveforms were collected, a precise measurement of the distance between the Doppler probes was recorded using a scientific caliper. The transit time between Doppler sites was determined using the foot‐to‐foot method with WinDAQ Waveform Browser (DataQ Instruments). Aortic PWV was calculated as the distance traveled divided by transit time.

### 2.7 Ex vivo vasodilatory function

To examine EDD, mesenteric arteries were dissected, cleared of surrounding tissue, and cannulated in the stage of a pressure myograph (DMT Inc.). Arteries were pre‐constricted with 2 μM phenylephrine, and EDD and the contribution of nitric oxide (NO) to dilation were measured in response to the cumulative addition of acetylcholine (10^−9^–10^−4^ M) in the presence and absence of the NO synthase inhibitor, L‐NAME (0.1 mmol/L, 30 min), as described previously (Islam et al., 2020; Islam, Cai, et al., 2023). NO bioavailability was assessed by subtracting acetylcholine‐induced maximal dilation in the presence of L‐NAME from acetylcholine‐induced maximal dilation in the absence of L‐NAME. To examine the superoxide‐mediated suppression of EDD, acetylcholine dose responses in the absence and presence of L‐NAME were performed in mesenteric arteries after incubation with the superoxide dismutase mimetic, TEMPOL (1 mM, 1 h). Endothelium‐independent dilation was assessed by measuring vasodilation in response to the cumulative addition of the inorganic NO donor sodium nitroprusside (10^−10^–10^−4^ M; Islam et al., 2020). Vessel diameters were measured using MyoView software (DMT Inc.). All dose–response data are presented as percent of possible dilation after preconstriction to phenylephrine. Arteries falling to achieve ≥20% pre‐constriction were excluded.

### 2.8 Superoxide production

Production of superoxide was measured by electron paramagnetic resonance (EPR) spectrometry using the spin probe 1‐hydroxy‐ 3‐methoxycarbonyl‐2,2,5,5‐tetramethylpyrrolidine (CMH, Alexis Biochemicals), as described previously (Lesniewski et al., 2017). Briefly, stock solutions of CMH were prepared in ice‐cold deoxygenated Krebs–HEPES buffer (mmol/L: NaCl, 99.01, KCl 4.69, CaCl_2_ 2.50, MgSO_4_1.20, K_2_HPO_4_1.03, NaHCO_3_ 25.0, glucose 11.10, and Na‐HEPES 20.00; pH 7.4), containing 0.1 mmol/L diethylenetriamine‐penta‐acetic acid and 5 μmol/L sodium diethyldithiocarbamate and pretreated with Chelex (Sigma) to minimize autooxidation of the spin probe. Two‐millimeter carotid artery rings were washed once in PSS and again in modified Krebs–HEPES buffer. Rings were then incubated for 60 min at 37°C in 180 μL Krebs–HEPES buffer containing 0.5 mmol/L CMH and analyzed immediately on an MS300 X‐band EPR spectrometer (Magnettech). Instrument settings were as follows: microwave frequency 9.83 GHz, centerfield 3480 G, sweep 80 G, modulation amplitude 3.3 G, microwave power 40 mW, microwave attenuation 7, and receiver gain 30. A total of six sweeps were conducted lasting 8.7 s per sweep. The running average of the six sweeps was collected with the double integration (area under and over the baseline) of the triplet used to display the magnitude of the signal. The magnitude of this signal directly relates to the amount of superoxide that has been trapped by the CMH.

### 2.9 Metabolic testing

We examined metabolic function using a battery of metabolic assessments such as glucose‐, insulin‐, pyruvate‐, and lipid‐ tolerance tests (GTT, ITT, PTT, and LTT) as well as a glucose‐stimulated insulin secretion (GSIS) assay. GTT, ITT, PTT, and GSIS assays were performed as described previously (Islam, Tuday, et al., 2023; Trott et al., 2021). Briefly, mice were fasted for 4–6 hours in the morning. Baseline blood glucose was measured using a Precision Xceed Pro Glucometer in blood collected via a tail nick. Mice were injected with either glucose (2 g/kg body mass, ip, GTT), insulin (1 U/kg body mass, ip, ITT), or sodium pyruvate (2 g/kg body mass, ip, PTT). Blood glucose was assessed at 15, 30, 60, 90, and 120 min after injection. For the GSIS assay, 20–25 μL blood was collected before and 15 min after the glucose injection and plasma was separated using a benchtop centrifuge. Baseline and glucose‐stimulated plasma insulin were measured using commercially available mouse insulin enzyme‐linked immunosorbent assay kit (Chrystal Chem) according to the manufacturer's protocol. For the LLT, mice were fasted overnight, and baseline blood was collected in the morning via tail nick. Twenty percent intralipid solution was administered (15 μL/g body mass, oral gavage), and blood was collected 1, 2, and 3 h after intralipid administration. Plasma was separated using a benchtop centrifuge and plasma triglycerides were measured using a commercial kit (Thermo Fisher) according to the manufacturer's protocol.

### 2.10 Plasma lipids

Total cholesterol, total triglycerides, and LDL and HDL concentrations were measured using dedicated on‐board reagents from Abbott Laboratories (Cat#7D62‐21, 7D74‐21, 1E31‐20, and 3k33‐22).

### 2.11 RT‐qPCR

Total mRNA was isolated using RNeasy Mini Kit (Qiagen) according to the manufacturer's protocol. mRNA was converted into cDNA using QuantiTect Reverse Transcription Kit (Qiagen) according to the manufacturer's protocol. Quantitative PCR was performed on 96‐well plates using SsoFast EvaGreen Supermixes (Bio‐Rad) with the Bio‐Rad CFX™ Real‐Time System. Gene expression was normalized to 18s, and fold change was calculated using the 2^−ΔΔCt^ method. Primer sequences are provided in the Table S3.

### 2.12 Statistics

Statistical analyses were performed using GraphPad Prism software, and data are presented as mean ± SEM with individual data points where appropriate. Data were tested for normality before group differences were assessed. All data passed the normality test. Most of the differences between age, sex, and genotypes were assessed by two‐way ANOVA or student's *t* test. Differences in vasodilation as well as GTT, ITT, PTT, LTT and GSIS were assessed by repeated measure ANOVA. Statistical significance was set at *p* < 0.05.

## 3 RESULTS

### 3.1 Aortic p‐S6K expression is augmented by advanced age and endothelial cells are the primary contributor

**FIGURE 1:**
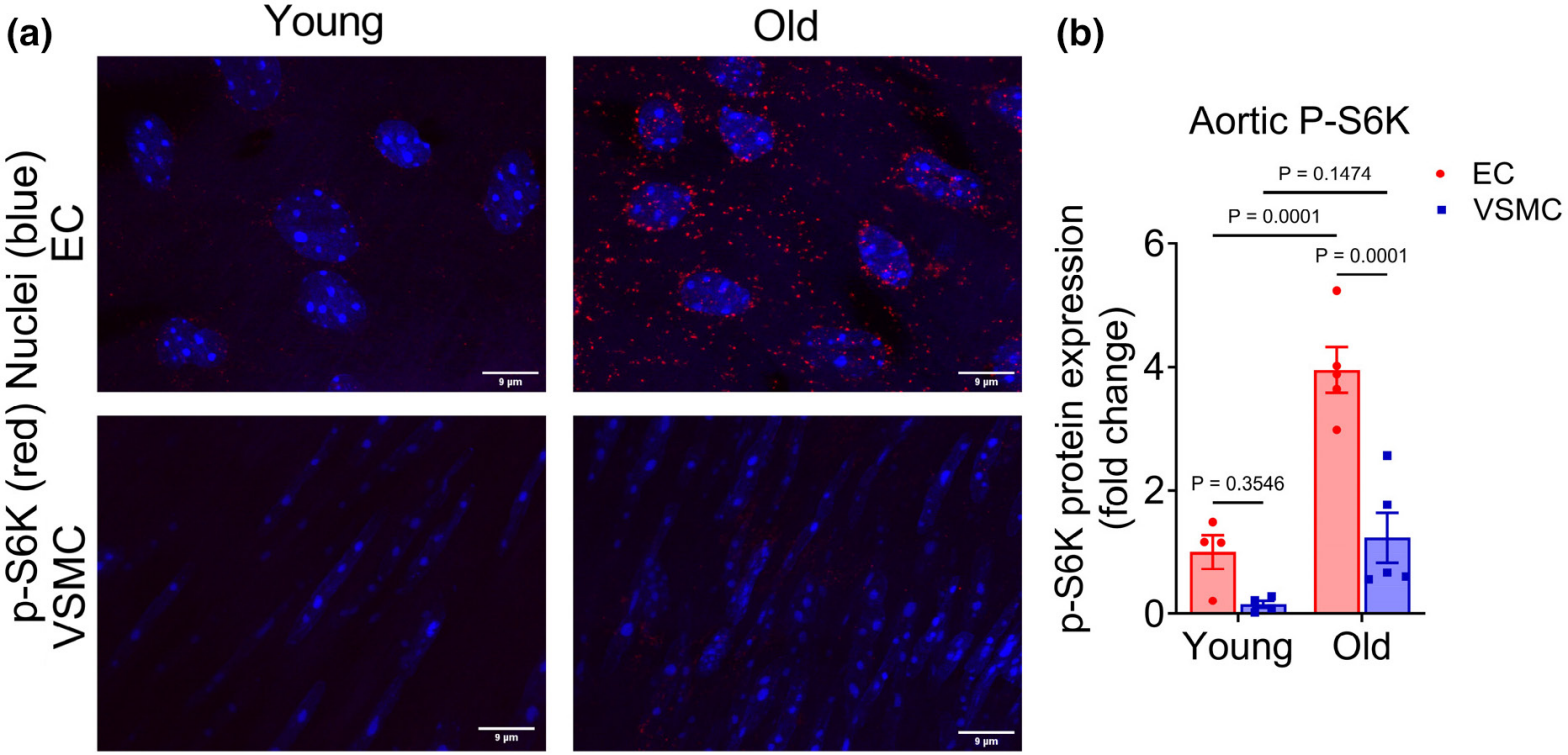
Endothelial cells are the primary contributors to arterial p‐S6K, which is markedly augmented by aging. (a) Representative images of p‐S6K and nuclear staining in aortic endothelial cells (ECs) and vascular smooth muscle cells (VSMCs) of young and old mice and (b) quantification of EC and VSMC p‐S6K expression. Data are shown as mean ± SEM with individual data points. *N* = 4–5/group. Differences were assessed using two‐way ANOVA with Tukey's post hoc test.

We first sought to examine the relative contribution of endothelial cells and vascular smooth muscle cells to total arterial mTOR activity and the impact of advancing age on this activation. To do so, we assessed the expression of phosphorylated S6K, a downstream effector of the mTOR signaling pathway, in en‐face preparations of aortas from young and old mice. We found that expression of p‐S6K was higher in the endothelial cells compared with vascular smooth muscle cells (*p* = 0.02; Figure 1a,b) in aortas of young mice. Advancing age resulted in an increase in p‐S6K expression in both endothelial cells and vascular smooth muscle cells (both *p* ≤ 0.04; Figure 1a,b). Despite this age‐related increase in both cell types, the contribution of endothelial cells to total arterial p‐S6K remained 3–4 fold higher compared with vascular smooth muscle cells (*p* ≤ 0.001; Figure 1a,b). Taken together, these results suggest that arterial mTOR signaling increases with advancing age, and endothelial cells are the key driver of this age‐related elevation.

### 3.2 Administration of tamoxifen attenuates mTOR expression in the endothelial cells of mTOR^f/f^ Cdh5 Cre + mice

To verify Cdh5 Cre‐mediated gene recombination and subsequent reductions in mTOR expression, we assessed protein expression in primary lung endothelial cells from tamoxifen‐treated *chd5*‐cre^+^ *mTOR* ^f/f^ (KO) and *chd5*‐cre^−^ *mTOR* ^f/f^ (WT) mice. We found a 45–50% reduction in mTOR in ECs from KO compared with WT mice (*p* = 0.002; Figure S1C,D). Two weeks after the administration of tamoxifen, aortic endothelial mTOR protein, assessed by immunofluorescence on en‐face preparations of aorta, was reduced by 60–70% in the KO compared with WT control mice (*p* = 0.006; Figure S1E,F).

### 3.3 Reduction in endothelial mTOR did not alter body or organ mass

**FIGURE 2:**
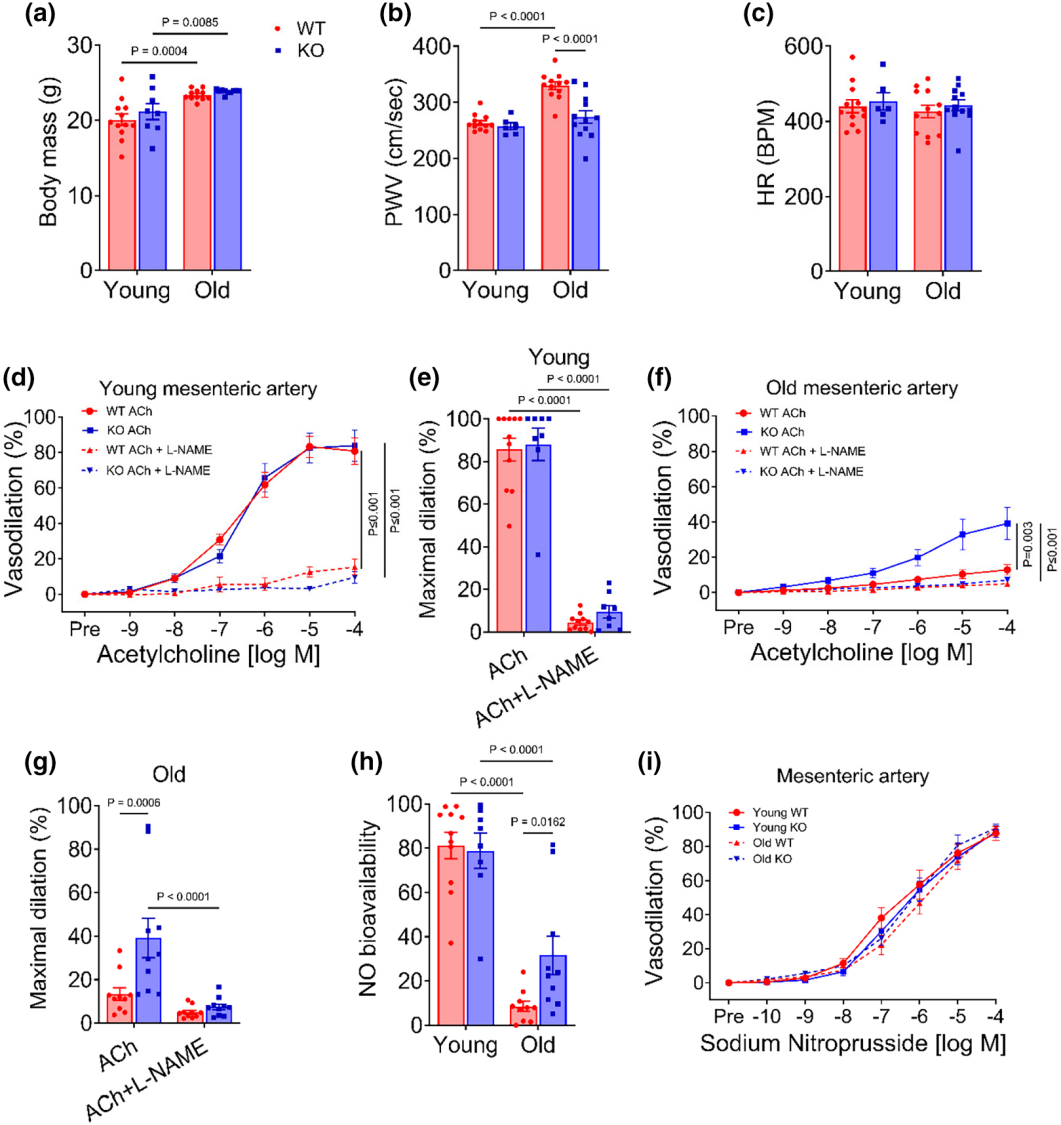
Reduction in EC mTOR reverses the age‐related increase in aortic stiffness and impairments in endothelium‐dependent dilation. (a) Body mass, (b) aortic pulse wave velocity (PWV), (c) heart rate, (d–g) concentration‐response curves and maximal dilation to acetylcholine (ACh) in the absence‐ and presence‐ of nitric oxide (NO) synthase inhibitor, L‐NAME in mesenteric arteries, (h) NO bioavailability in mesenteric arteries, and (i) concentration‐response curves for the endothelium‐independent vasodilator, sodium nitroprusside in young and old wildtype (WT) and knockout (KO) mice. Data are shown as mean ± SEM with individual data points. *N* = 6–12/group. Two‐way ANOVA and RM‐ANOVA were performed to assess group differences with Tukey's post hoc test where appropriate.

Two months after the induction of endothelial mTOR deletion, we did not observe any difference in body mass between WT and KO in young mice (*p* = 0.14; Figure 2a). Although aging increased the body mass in both WT and KO mice (*p* < 0.001), we did not observe any difference between groups in old mice (*p* = 0.70; Figure 2a). Likewise, we did not find any difference in organ mass including liver, skeletal muscle, adipose tissue, liver, heart, kidney, and spleen between old WT and KO mice (all *p* ≥ 0.23; Table S1).

### 3.4 Reduction in endothelial mTOR reverses age‐related arterial stiffening and impairments in EDD

To assess arterial function, we examined arterial stiffness and EDD, two major hallmarks of arterial dysfunction that precede clinical cardiovascular diseases. In young mice, EC mTOR reduction did not alter PWV, a measure of arterial stiffness (*p* = 0.25; Figure 2b). Aging resulted in an elevated aortic PWV (*p* = 0.005), indicating higher stiffness in WT mice, and deletion of EC mTOR reversed aortic stiffening in old mice (*p*<0.001; Figure 2b). We did not observe any difference in anesthetized heart rate (HR) between genotypes or ages (*p* ≥ 0.46; Figure 2c), suggesting that the differences in arterial stiffness are independent of changes in HR. In young mice, EDD to acetylcholine was not different between KO and WT mice (*p* = 0.95, Figure 2d,e). Vasodilation was reduced in the presence of the NO synthase inhibitor, L‐NAME, in both KO and WT young mice (*p* ≤ 0.001), but no difference was found between groups (*p* = 0.89; Figure 2d,e). EDD to acetylcholine was lower in mesenteric arteries of old mice compared with young (*p* = 0.002; Figure 2d–g). Reduction in EC mTOR markedly improved EDD (*p* = 0.008; Figure 2f) and increased NO bioavailability (*p* = 0.012; Figure 2h) in old mice. Dilation to the inorganic NO donor, sodium nitroprusside was not different either between genotypes or ages (*p* = 0.92; Figure 2i), indicating that alterations in EDD are independent of vascular smooth muscle cell responsiveness to NO. Taken together, these results demonstrate that a reduction in endothelial mTOR does not alter arterial stiffness or EDD in young mice but ameliorates these expressions of arterial dysfunction in old mice.

### 3.5 Improvements in vascular function are accompanied by suppressed oxidative stress and gene expression of senescence, inflammation, and arterial remodeling markers

**FIGURE 3:**
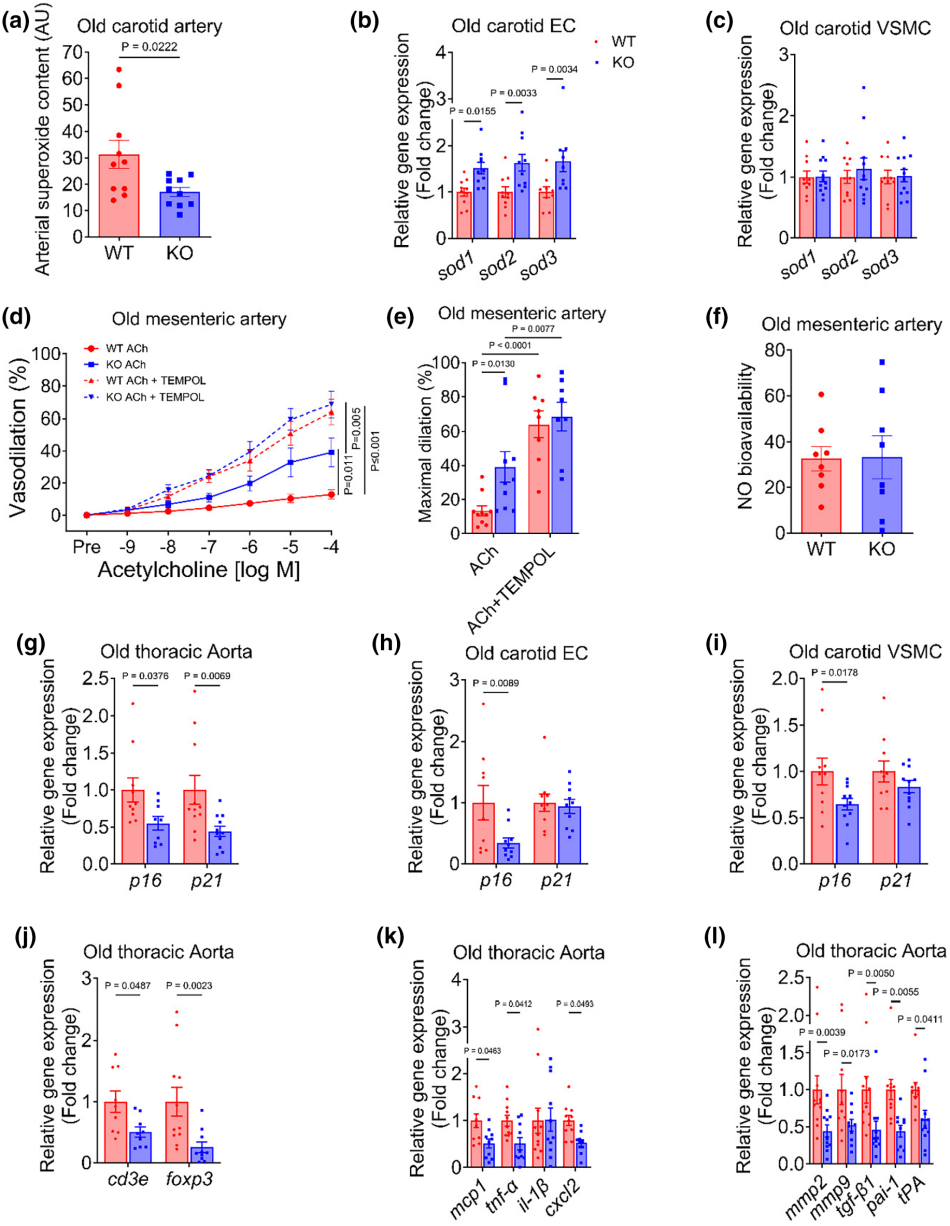
Improved arterial function in old EC mTOR knockout mice is accompanied by suppressed oxidative stress, senescence, and inflammation. (a) Carotid artery superoxide content, (b, c) gene expression of superoxide dismutase 1, 2 and 3 (sod1, sod2, and sod3) in endothelial cell‐ and vascular smooth muscle cell‐enriched carotid artery lysates, (d) concentration‐response curves, (e) maximal dilation to acetylcholine (ACh) in the absence and presence of superoxide scavenger, TEMPOL, (f) nitric oxide bioavailability in the presence of TEMPOL, (g–i) gene expression of p16 and p21 in thoracic aorta as well as endothelial cell‐ and vascular smooth muscle cell‐enriched carotid artery lysates. (j–l) gene expression of total (cd3e) and regulatory (foxp3) T cells, inflammatory cytokines, and matrix proteins in the thoracic aorta. Data are shown as mean ± SEM. *N* = 8–10/group. Independent Student's *t* test and RM‐ANOVA were performed to assess group differences.

Elevated oxidative stress, senescence, and inflammation are major underlying causes of age‐related arterial dysfunction. Therefore, we sought to examine the impact of reducing endothelial mTOR on these macro‐mechanistic processes. We found that the total content of superoxide was lower in carotid arteries from old KO compared with WT mice (*p* = 0.01, Figure 3a). Expression of the antioxidant genes, superoxide dismutases, *sod1*, *sod2*, and *sod3* were higher in endothelial‐enriched carotid artery effluents (all *p* ≤ 0.01, Figure 3b). We did not find any difference in *sod1*, *sod2*, and *sod3* gene expression in vascular smooth muscle cell‐enriched carotid artery lysates (all *p* ≥ 0.28, Figure 3c). To determine whether superoxide suppressed EDD, we examined ACh‐mediated vasodilation in the presence of a superoxide scavenger, TEMPOL. TEMPOL treatment increased EDD in arteries from both KO and WT mice, although the magnitude of improvement was greater in WT mice compared with KO mice (all *p* ≤ 0.021, Figure 3d,e). Likewise, in the presence of TEMPOL, NO bioavailability was similar between groups (Figure 3f). Collectively, these results indicate that a reduction in endothelial mTOR suppresses oxidative stress in old mice, thereby increasing NO bioavailability. To examine whether reduced mTOR lowered the burden of senescence, we assessed gene expression for *p16* and *p21* in thoracic aorta as well as EC‐ and VSMC‐enriched carotid artery lysates. *p16* and *p21* gene expressions were lower in the thoracic aorta from old KO compared with WT mice. Furthermore, *p16*, but not *p21*, gene expression was lower in both EC‐ and VSMC‐enriched carotid artery lysates from old KO compared to WT mice (all *p* ≤ 0.03, Figure 3g–i). We also assessed the expression of T‐cell genes (*cd3e* and *foxp3*), senescence‐associated secretory phenotype (SASP) genes (*mcp1*, *tnf‐α*, *il‐1β*, and *cxcl2*), and genes that are associated with arterial remodeling (*mmp2*, *mmp9*, *tgf‐β1*, *pai‐1*, and *tPA*) and found them to be lower in aorta from old KO compared with WT mice (all *p* ≤ 0.04, Figure 3j–l). Taken together, these findings indicate that improvements in arterial function are concomitant with suppressed oxidative stress, as well as reduced gene expression for senescence, inflammatory, and arterial remodeling markers.

### 3.6 Reduction in endothelial mTOR improves systemic metabolic function

**FIGURE 4:**
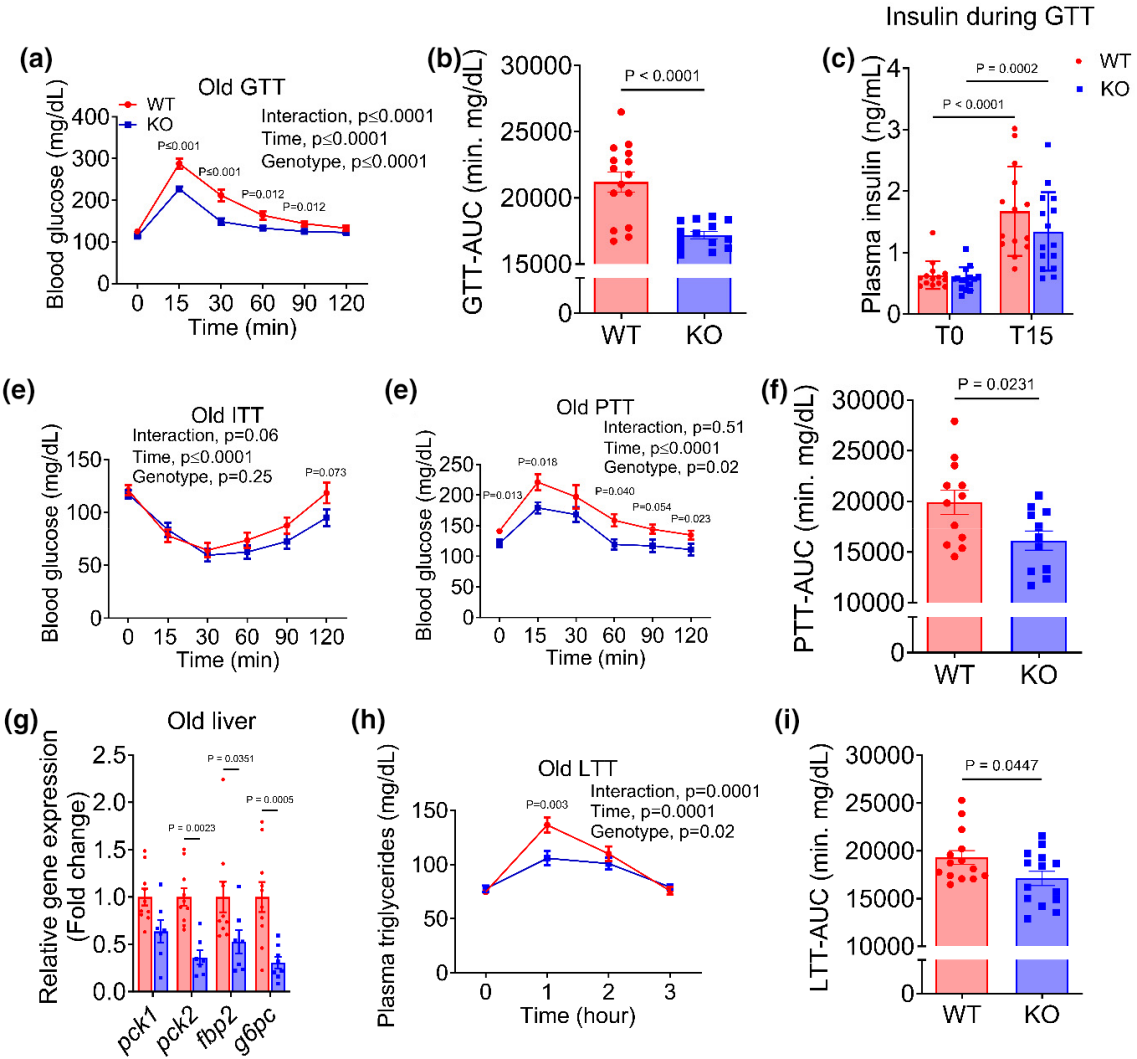
Reduction in EC mTOR improves systemic metabolic function in old mice. (a) Time‐response curves and (b) area under the curve during a glucose‐tolerance test (GTT), (c) baseline and glucose‐stimulated (15 min) plasma insulin, (d) time‐response curves during an insulin tolerance test (ITT), (e) time‐response curves and (f) area under the curve during a pyruvate tolerance test (PTT), (g) hepatic expression of gluconeogenic genes, pck1, pck2, fbp2, and g6pc, (h) time‐response curve and (i) area under the curve during an intralipid‐tolerance test. Data are shown as mean ± SEM. *N* = 11–15/group. Independent Student's *t* test and RM‐ANOVA were performed to assess group differences.

Emerging evidence suggests that endothelial cells play a critical role in systemic metabolic function. Here, we tested the hypothesis that improvements in endothelial function will ameliorate metabolic function in old mice but will not impact young mice. We found that glucose tolerance was not different between KO and WT young mice (*p* = 0.33, Figure S3A), and insulin sensitivity was modestly improved in young KO compared with WT littermates (*p* = 0.03, Figure S3B). In old mice, both the time‐response curve and the area under the curve during a GTT were lower in KO compared with WT littermates (all *p* ≤ 0.001; Figure 4a,b), indicating an improvement in glucose tolerance. To examine the underlying mechanisms of improved glucose tolerance in old mice, we performed GSIS, ITT and PTT. Baseline and glucose‐stimulated insulin secretion were not different between groups (all *p* ≥ 0.20; Figure 4c). Likewise, we did not find any difference in insulin sensitivity between KO and WT mice (*p* = 0.25, Figure 4d). However, both the time‐response curve and the area under the curve during a PTT were lower in KO compared with WT mice (all *p* ≤ 0.02; Figure 4e,f), suggesting attenuated hepatic gluconeogenesis. Moreover, expression of gluconeogenic genes (*pck1*, *pck2*, *fbp2*, *and g6pc*) was lower in KO compared with WT mice (all *p* ≤ 0.04, Figure 4g). Collectively, these results demonstrate that improvement in glucose tolerance in old mice occurred due to suppressed hepatic gluconeogenesis but was independent of pancreatic beta cell function or insulin sensitivity. To examine whether reductions in endothelial mTOR improve lipid metabolism, we assessed plasma lipid profiles as well as performed a LTT. We did not find any difference in total cholesterol, low−/very low‐, and high‐density lipoproteins or triglycerides (all *p* ≥ 0.24; Figure S4). However, the time‐response curve and the area under the curve during the LTT were lower in old KO compared with WT mice (all *p* ≤ 0.02; Figure 4h,i), suggesting an improvement in triglyceride tolerance. Taken together, these results provide compelling evidence that reducing endothelial mTOR improves systemic metabolic function in old mice.

### 3.7 Improvements in systemic metabolic function are concomitant to attenuated expression of markers of senescence and inflammatory genes

**FIGURE 5:**
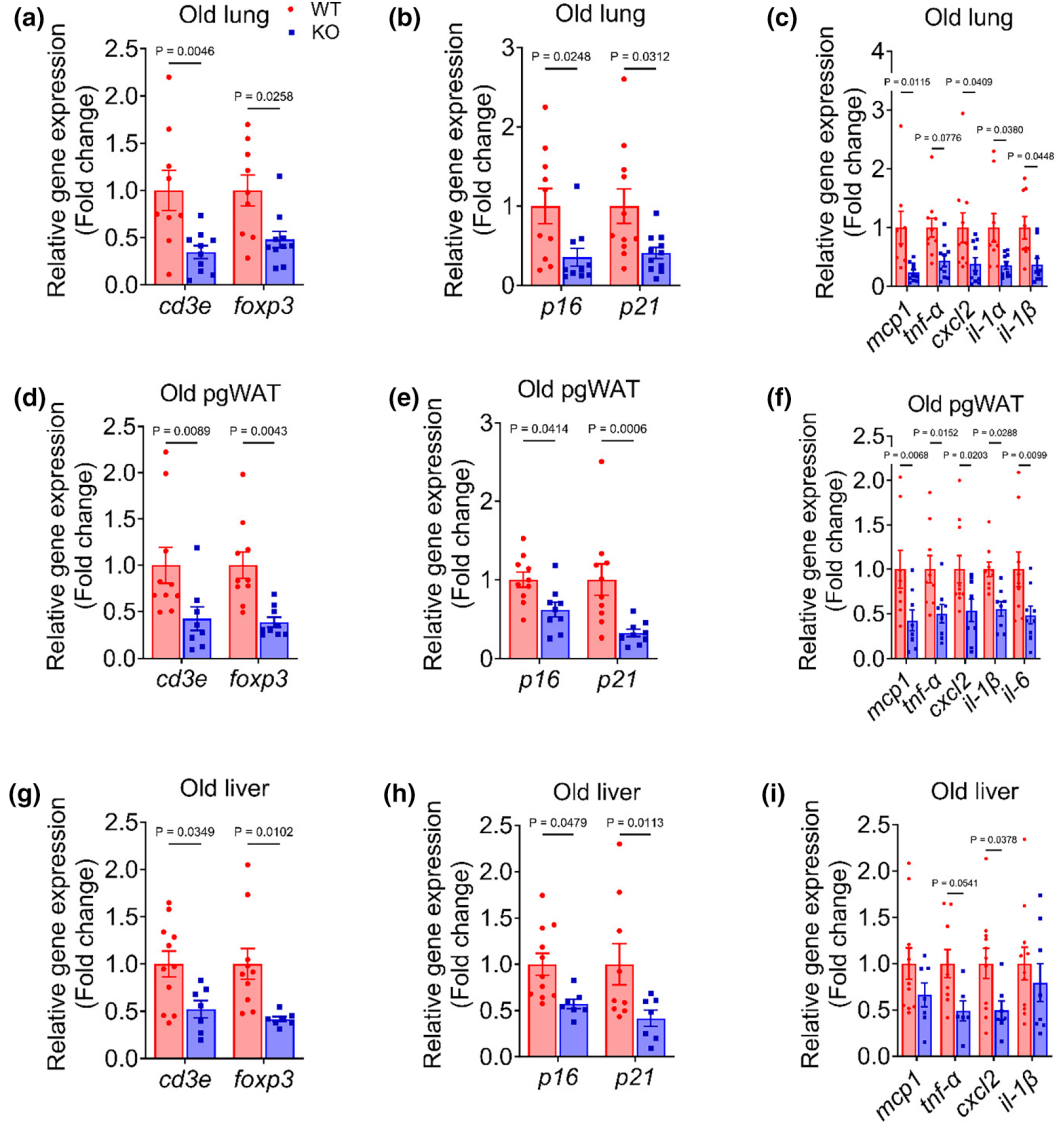
Reduction in EC mTOR attenuates hallmarks of aging across multiple organs. Expression of genes that are indicative of tissue T cell infiltration (cd3e and foxp3), senescent burden (p16 and p21), and inflammation (tnf‐α, il‐6, il‐1β, mcp1, and cxcl2) in (a–c) lung, (d–f) perigonadal adipose tissue (pgWAT) and (g–i) liver of old wildtype (WT) and knockout (KO) mice. Data are shown as mean ± SEM. *N* = 7–12/group. Independent Student's *t* test was performed to assess group differences.

Endothelial dysfunction is a major driver of multi‐organ dysfunction. Therefore, we examined whether the improvement in endothelial function in old mTOR KO mice was concomitant with an attenuation of hallmarks of aging such as senescence and inflammatory burden in endothelial‐rich and/or metabolically active tissues such as lung, perigonadal adipose tissue (pgWAT) and liver. To do so, we assessed gene expression of T cells (*cd3e* and *foxp3*), senescence (*p16*, *p21*), and inflammatory (*mcp1*, *tnf‐α*, *cxcl2*, *il‐1β*, *il‐6*) markers. Expression of these genes was lower in lung, pgWAT, and liver from KO compared with WT mice (all *p* ≤ 0.05; Figure 5), suggesting that reductions in endothelial mTOR attenuate tissue inflammation via attenuated burden of immune cells, senescence, and inflammation.

## 4 DISCUSSION

In this study, we found that in young mice, endothelial cells are the major contributor to the arterial expression of p‐S6K, a downstream target of mTOR. While advancing age increases p‐S6K expression both in endothelial and vascular smooth muscle cells, in old mice, p‐S6K expression is significantly higher in endothelial compared with vascular smooth muscle cells. A reduction in endothelial cell‐specific mTOR reverses age‐related increases in arterial stiffness and rescues impairments in EDD but does not alter these functions in young mice. In old mice, the improvement in arterial function is accompanied by suppressed oxidative stress, senescence, and inflammation in arteries. In young mice, endothelium‐specific mTOR reduction does not alter glucose tolerance but modestly improves insulin sensitivity. In old mice, a reduction in endothelial mTOR does not alter baseline plasma lipids, but improves lipid and glucose tolerance and suppresses hepatic gluconeogenesis. The systemic improvement in metabolic function in old mice is concomitant with attenuated expression of markers of senescence and senescence‐associated inflammatory genes in the lung, liver, and perigonadal white adipose tissue. Taken together, this study demonstrates that endothelial mTOR plays a critical role in age‐related arterial and metabolic dysfunction. These results shed light on how systemic mTOR inhibition may improve age‐related arterial and metabolic dysfunction and provide direct evidence that endothelial cells play a critical role in this interplay. These findings also support the exploration of endothelial mTOR as a therapeutic target in age‐related cardiovascular and metabolic diseases.

Although pharmacological inhibition of mTOR has been demonstrated to extend murine lifespan (Harrison et al., 2009; Miller et al., 2011), whether mTOR signaling pathway is elevated with aging remains unclear. In this study, we demonstrate that p‐S6K, a downstream effector of the mTOR signaling pathway, increases with aging both in endothelial cells and vascular smooth muscle cells. Aging did not alter mTOR activation in muscle, adipose tissue, liver, and heart of the C57BL/6J mice from the National Institute on Aging colony (Baar et al., 2016). This observation led to the argument that in order to receive the beneficial effects of mTOR inhibition in advanced age, mTOR signaling does not necessarily need to increase with aging (Baar et al., 2016; Blagosklonny, 2009). However, in agreement with the present study, we and others have shown that unlike most tissues, aortic p‐S6K does increase with aging (Baar et al., 2016; Donato et al., 2013; Lesniewski et al., 2017; Rice et al., 2005). Furthermore, here we demonstrate that while arterial mTOR signaling is elevated with aging, endothelial cells are the key contributors to this age‐related augmentation. Thus, our findings challenge the idea that mTOR activation does not change with advancing age and suggest that endothelial mTOR may play a critical role in mediating the benefits of systemic mTOR inhibition in old age.

Arterial stiffening and impairments in EDD are hallmarks of age‐related vascular dysfunction that precede and predict overt cardiovascular diseases (Donato et al., 2018; Islam et al., 2021). We have previously demonstrated that lifelong calorie restriction attenuates arterial stiffness and improves EDD in old mice and that this was accompanied by suppressed mTOR activation (Donato et al., 2013). In a subsequent study, we provided evidence that systemic mTOR inhibition using rapamycin lowers arterial stiffness and improves EDD in old mice (Lesniewski et al., 2017). In humans, administration of the immunosuppressant drug sirolimus, which works via mTOR inhibition, attenuates arterial stiffness in middle‐aged renal transplant recipients (Joannidès et al., 2011). Collectively, these previous studies indicate that mTOR inhibition exerts beneficial effects on arterial function with advancing age. However, a key limitation of these studies was that the tissues or organs mediating the beneficial effects of mTOR inhibition remained unknown. The present study indicates that a reduction in mTOR signaling in endothelial cells per se is sufficient to rescue age‐related arterial dysfunction.

An elevated burden of cellular senescence, inflammation, and oxidative stress are the major underlying causes of age‐related arterial dysfunction (Donato et al., 2018; Islam et al., 2021). Using human umbilical vein endothelial cells, it has been reported that elevated mTOR signaling promotes the development of, and remains elevated in, models of both replicative‐ and stress‐induced premature senescence (Rajapakse et al., 2011; Yang et al., 2018). Furthermore, senescent endothelial cells generate reactive oxygen species, which is suppressed by genetic and pharmacological mTOR inhibition (Rajapakse et al., 2011). Evidence also suggests that mTOR inhibition by rapamycin reduces stress‐induced premature senescence and inflammatory gene expression in human coronary artery endothelial cells (Sasaki et al., 2020). Similar roles of mTOR in senescence, inflammation, and oxidative stress have also been described in vascular smooth muscle cells, fibroblasts, and fibrosarcoma cell lines (Demidenko et al., 2009; Sung et al., 2018; Xia et al., 2017). Likewise, we have previously demonstrated that systemic rapamycin administration in old mice improves ex vivo vasodilation that was associated with reduced senescence and oxidative stress (Lesniewski et al., 2017). Taken together, evidence to date and findings from the present study suggest that suppression of senescence, inflammation, and oxidative stress may underlie the improvements in arterial function afforded by endothelial mTOR reduction.

Emerging evidence suggests that the vascular endothelium is a critical regulator of systemic metabolic function, although the mechanisms remain elusive (Graupera & Claret, 2018; Pi et al., 2018). While systemic mTOR inhibition has been demonstrated to extend lifespan in multiple model organisms and improve many age‐related diseases, this treatment regimen also induces glucose intolerance due to elevated insulin resistance and hepatic gluconeogenesis (Johnson et al., 2013). Another common adverse metabolic side effect of systemic mTOR inhibition is dyslipidemia (Claes et al., 2012; Johnson et al., 2013). These side effects hinder the routine clinical application of systemic mTOR inhibitors as anti‐aging interventions in humans (Johnson et al., 2013). In the current study, we did not find any difference in plasma lipid profiles such as triglycerides, total‐, low‐, and high‐density cholesterols in old mice. Taken together, our findings suggest that endothelial‐specific mTOR reduction not only helps avoid any detrimental metabolic side effects but also can be an effective strategy to improve metabolic function in old age. Future studies are warranted to develop strategies to deliver pharmacological mTOR inhibitors such as rapamycin specifically to endothelial cells. An emerging class of lipid nanoparticle‐based local drug delivery techniques can be harnessed in this endeavor (Khare et al., 2023).

The underlying mechanisms for the improvement in systemic metabolic function in old mice afforded by endothelial mTOR reduction could be multifaceted. Evidence exists that endothelial cell senescence and telomere dysfunction play a causal role in systemic metabolic dysfunction (Barinda et al., 2020; Yokoyama et al., 2014). For example, endothelial cell‐specific inhibition of inflammatory mediator, nuclear factor‐κB, prevented obesity‐ and age‐related metabolic dysfunction due to attenuated vascular senescence, inflammation, and oxidative stress (Hasegawa et al., 2012). Likewise, endothelial‐specific deletion of the tumor suppressor gene, p53, reduced diet‐induced obesity, glucose intolerance, and insulin resistance (Yokoyama et al., 2014). It has also been shown that conditioned media from senescent endothelial cells induced senescence and inflammatory gene expression as well as reduced insulin signaling in 3T3‐L1 adipocytes, suggesting a crosstalk between endothelium and extravascular tissues via secreted factors (Barinda et al., 2020). Thus, the observed beneficial metabolic effects and attenuated burden of senescence and inflammation in lung, liver, and adipose tissue after a reduction in endothelial mTOR are potentially mediated through suppressed endothelial cell senescence.

## 5 CONCLUSION, LIMITATIONS, AND FUTURE DIRECTIONS

In summary, findings from this study support the conclusion that age‐related increases in endothelial cell mTOR signaling is a reversible cause of arterial and metabolic dysfunction. Attenuated senescence, inflammation, and reactive oxygen species are key underlying causes of improved arterial and metabolic function afforded by a reduction in endothelial mTOR. Importantly, unlike systemic mTOR inhibition, endothelial‐specific reductions in mTOR lead to either benign or beneficial effects on metabolic function. While this study advances our understanding of endothelial mTOR signaling in age‐related arterial and metabolic dysfunction, it creates many open questions that can be pursued in future research. For example, the mechanisms by which endothelial mTOR deletion mediates these beneficial effects require further elucidation. The effects of endothelial mTOR reduction can be tested in other age‐related dysfunction and diseases with an endothelial and/or arterial origin such as atherosclerosis, heart diseases, retinopathy, nephropathy, and cancer. Moreover, other benefits of systemic mTOR inhibition by rapamycin such as lifespan extension, improved immune function, and attenuation of neurodegenerative diseases can be tested using this endothelial‐specific mTOR deletion mouse model (Johnson et al., 2013; Liu & Sabatini, 2020). Recent technological advances in local drug delivery (Friedman et al., 2013) may foster the development of mTOR inhibitors that will selectively target endothelial cells. The development of such a drug may open a new therapeutic avenue with high translational value.

## AUTHOR CONTRIBUTIONS

MTI and LAL were involved in all aspects of the study including experimental design, data collection, analyses and manuscript preparation. SAH, TD, SIB, RCB, JK, JRT, DRM, and AJD participated in data collection and/or manuscript preparation.

## 7 FUNDING INFORMATION

This work was supported by the National Institutes of Health Awards R01 AG048366 (LAL), R01 AG050238 (AJD), R01 AG060395 (AJD), F31AG076312 (SIB), and Veteran's Affairs Merit Review Award I01 BX004492 (LAL) from the US Department of Veterans Affairs Biomedical Laboratory Research and Development Service. The contents do not represent the views of the US Department of Veterans Affairs, the National Institutes of Health, or the US Government.

## CONFLICT OF INTEREST STATEMENT

The authors declare no competing interests.

## Supporting information


Appendix S1.

## Data Availability

The data that support the findings of this study are available from the corresponding author upon reasonable request.
