# Supplementary material for: Endothelial cell‐specific reduction in mTOR ameliorates age‐related arterial and metabolic dysfunction
Source: Aging Cell. 2023 Nov 28;23(2):e14040. doi: 10.1111/acel.14040 (PMC10861194; doi:10.1111/acel.14040)
Supplement: Supplementary file 1 — Appendix S1. [file ACEL-23-e14040-s001.docx]

**Supplementary Figure 1: Generation and validation of endothelial cell-specific mTOR knockout mouse model.** (A, B) Generation and identification of endothelial cell (EC)-specific tamoxifen inducible mTOR knockout (KO) mouse model, the PCR product size of mTOR^f/f^ bands were 533 base pairs (bp), wildtype mTOR bands were 349 bp, and Cdh5Cre positive were 760 bp, Cdh5Cre negative mice did not have any PCR product/band (C, D) western blot images and quantification of mTOR and vinculin proteins from primary lung endothelial cells from wildtype (WT) and KO mice (N= 3/replicate, ECs were pooled from lungs of 4-5 mice). (E) Representative images of mTOR and nuclear staining in aortic ECs of WT and KO mice and (F) quantification of EC mTOR expression normalized to WT mean. Data are shown as mean ± SEM with individual datapoint. N=3-5/group. Independent Student’s t test was performed to assess group difference.


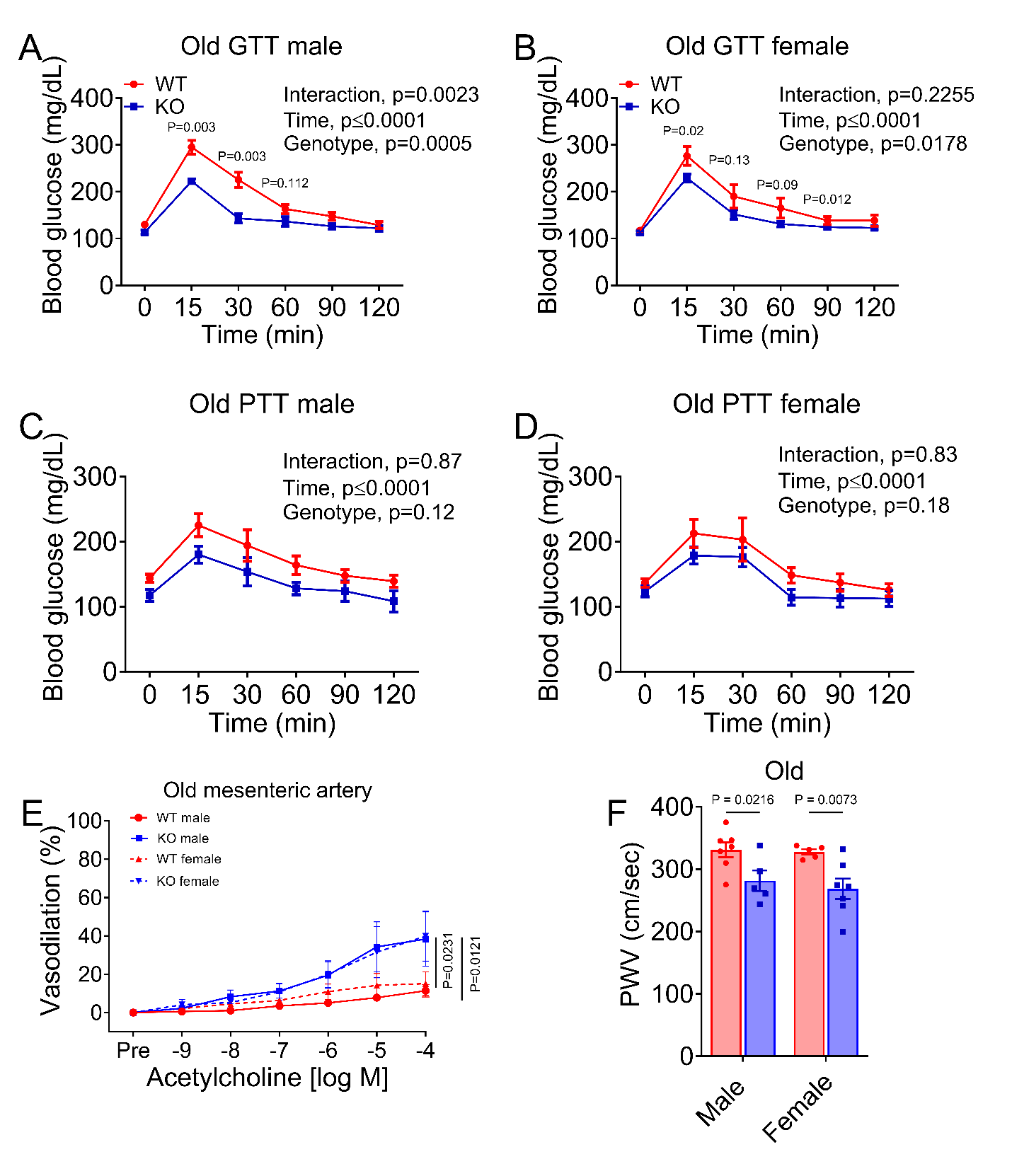


**Supplementary Figure 2: Effects of endothelial mTOR reduction on metabolic and arterial function in male and female mice.** (A, B) Blood glucose response curves of old wildtype (WT) and knockout (KO) male and female mice during a glucose tolerance test (GTT), (C, D) blood glucose response curves of old WT and KO male and female mice during a pyruvate tolerance test (PTT), (E) concentration-response curves for acetylcholine (ACh) of old WT and KO male and female mice, (F) pulse wave velocity (PWV) of old WT and KO male and female mice**.** Data are shown as mean ± SEM with individual datapoint. N=4-10/group. Two-way ANOVA and RM-ANOVA were performed to assess group differences with Tukey’s post hoc test where appropriate.


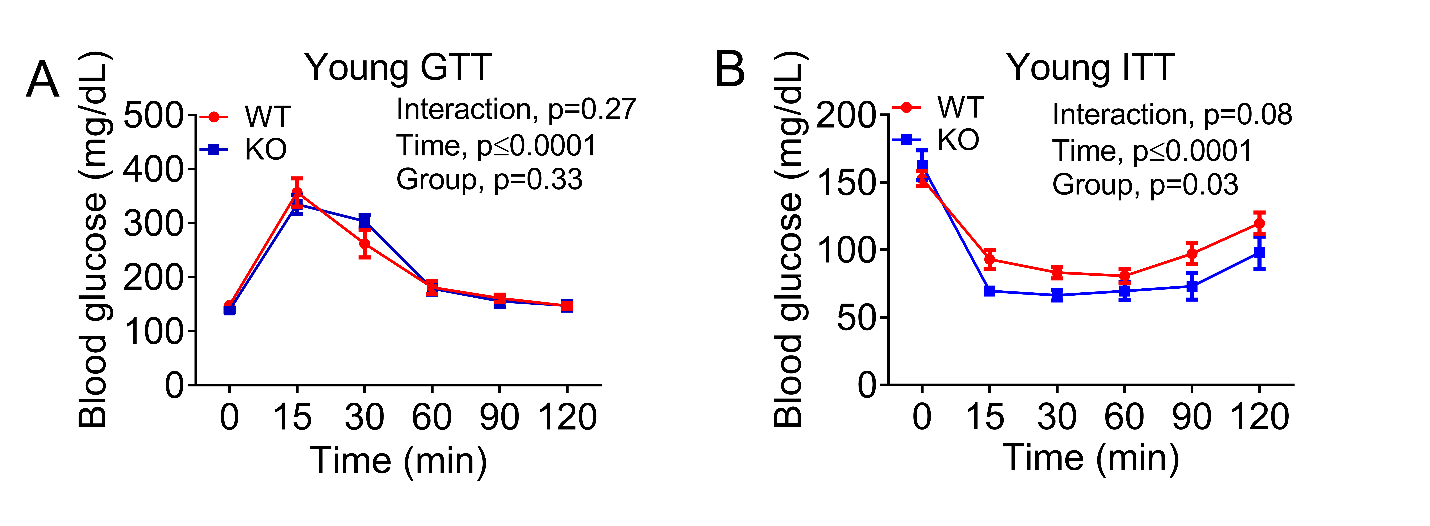


**Supplementary figure 3: Reduction in endothelial mTOR does not alter glucose tolerance or insulin sensitivity in young mice.** Time response curve during (A) glucose-tolerance test (GTT) and insulin tolerance test (ITT). Data are shown as mean ± SEM. N=8-10/group. Independent Student’s t test and RM-ANOVA were performed to assess group differences.


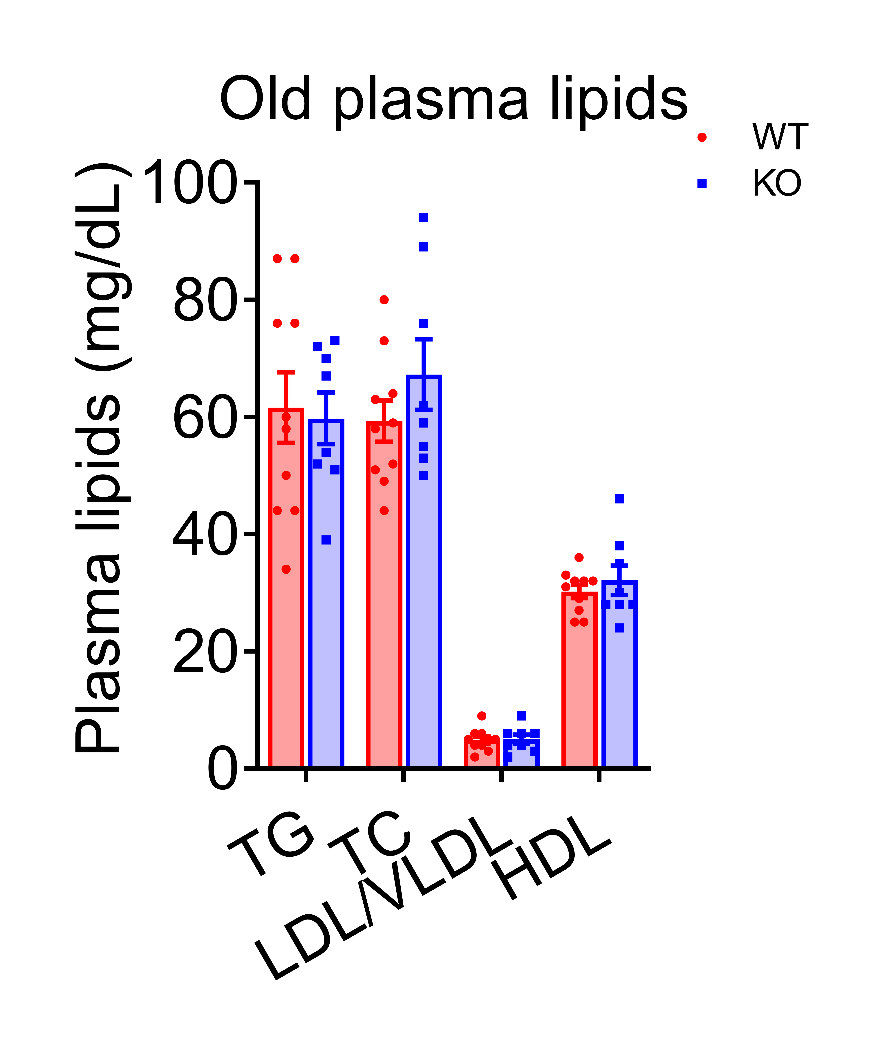


**Supplementary figure 4: Reduction in endothelial mTOR does not alter plasma lipids in old mice.** Plasma concentration of triglycerides (TG), total cholesterols (TC), low- and very low-density lipoproteins (LDL/VLDL), high density lipoproteins (HDL) from knockout (KO) and wildtype (WT) old mice. Data are shown as mean ± SEM. N=8-10/group. Independent Student’s t test was performed to assess group differences.

| **Supplementary table 1: Characteristics of old mice** | | | | | | | |
| --- | --- | --- | --- | --- | --- | --- | --- |
|  | WT | | | KO | | | p value |
| Age (mo) | 23.4 | ± | 0.2 | 23.8 | ± | 0.1 | 0.12 |
| Body mass (g) | 25.34 | ± | 1.25 | 26.19 | ± | 1.00 | 0.70 |
| Liver mass (g) | 1.50 | ± | 0.06 | 1.42 | ± | 0.07 | 0.53 |
| Gastrocnemius mass x 2 (mg) | 253 | ± | 16 | 280 | ± | 16 | 0.23 |
| Quadriceps mass x 2 (mg) | 261 | ± | 22 | 270 | ± | 20 | 0.85 |
| Soleus mass x 2 (mg) | 20 | ± | 2 | 20 | ± | 1 | 0.28 |
| WAT mass x 2 (mg) | 180 | ± | 43 | 145 | ± | 20 | 0.53 |
| Kidney mass x 2 (mg) | 452 | ± | 37 | 413 | ± | 14 | 0.30 |
| Heart mass (mg) | 167 | ± | 9 | 166 | ± | 10 | 0.61 |
| Spleen mass (mg) | 138 | ± | 17 | 137 | ± | 27 | 0.82 |
| Data are shown as mean ± SEM, N=8-10/group | | | | | | | |

| **Supplementary table 2: Primer sequences for genotyping** | | |
| --- | --- | --- |
| Genes | Forward | Reverse |
| mTOR | 5’-TTATGTTTGATAATTGCAGTTTTG-3’ | 5’-TTTAGGACTCCTTCTGTGACATA-3’ |
| Cdh5 | 5’-GCCTGCATTACCGGTCGATGCAACGA-3’ | 5’-GTGGCAGATGGCGCGGCAACACCATT-3’ |

| **Supplementary table 3: Primer sequences for qPCR** | | |
| --- | --- | --- |
| Genes | Forward | Reverse |
| *18s* | 5′-TAGAGGGACAAGTGGCGTTC-3′ | 5′-CGCTGAGCCAGTCAGTGT-3′ |
| *Sod1* | 5′-AACCAGTTGTGTTGTCAGGAC-3′ | 5′-CCACCATGTTTCTTAGAGTGAGG-3′ |
| *Sod2* | 5′-CAGACCTGCCTACGACTATGG-3′ | 5′-CTCGGTGGCGTTGAGATTGTT-3′ |
| *Sod3* | 5′-CCTTCTTGTTCTACGGCTTGC-3′ | 5′-TCGCCTATCTTCTCAACCAGG-3′ |
| *P16* | 5′-CGCAGGTTCTTGGTCACTGT-3′ | 5′-TGTTCACGAAAGCCAGAGCG-3′ |
| *P21* | 5′-CCTGGTGATGTCCGACCTG-3′ | 5′-CCATGAGCGCATCGCAATC-3′ |
| *Cd3e* | 5′-GACTATGAGCCCATCCGCAAA-3′ | 5′-TAGGACACGTGTTCACCAGGA-3′ |
| *Foxp3* | 5′-GGCCCTTCTCCAGGACAGA-3′ | 5′-GCTGATCATGGCTGGGTTGT-3′ |
| *Mcp1* | 5′-GCATCCACGTGTTGGCTCA-3′ | 5′-CTCCAGCCTACTCATTGGGATCA-3′ |
| *Tnf-α* | 5′-ATGAGAAGTTCCCAAATGGC-3′ | 5′-CTCCACTTGGTGGTTTGCTA-3′ |
| *Il-1α* | 5′-CGAAGACTACAGTTCTGCCATT-3′ | 5′-GACGTTTCAGAGGTTCTCAGAG-3′ |
| *Il-1β* | 5′-CACAGCAGCACATCAACAAG-3′ | 5′-GTGCTCATGTCCTCATCCTG-3′ |
| *Il-6* | 5′-CTGGGAAATCGTGGAAT-3′ | 5′-CCAGTTTGGTAGCATCCATC-3′ |
| *Cxcl2* | 5′-CCTGGTTCAGAAAATCATCCA-3′ | 5′-CTTCCGTTGAGGGACAGC-3′ |
| *Mmp2* | 5′-CAAGTTCCCCGGCGATGTC-3′ | 5′-TTCTGGTCAAGGTCACCTGTC-3′ |
| *Mmp9* | 5′-CTGGACAGCCAGACACTAAAG-3′ | 5′-CTCGCGGCAAGTCTTCAGAG-3′ |
| *Tgf-β1* | 5′-CTCCCGTGGCTTCTAGTGC-3′ | 5′-GCCTTAGTTTGGACAGGATCTG-3′ |
| *Pai-1* | 5′-GACACCCTCAGCATGTTCATC-3′ | 5′-AGGGTTGCACTAAACATGTCAG-3′ |
| *tPA* | 5′-TGACCAGGGAATACATGGGAG-3′ | 5′-CTGAGTGGCATTGTACCAGGC-3′ |
| *Pck1* | 5′-CCTGGAAGAACAAGGAGTGG -3′ | 5′-AGGGTCAATAATGGGGCACT-3′ |
| *Pck2* | 5′-CCCTATCACAAGGCAAGAGA -3′ | 5′-CCACTTCCCCTGTCCTATTT -3′ |
| *Fbp2* | 5′-GGGGGAAATATGTGGTTTGCT -3′ | 5′-TCCTCCGTGGTCTTTCTGTAAA -3′ |
| *G6pc* | 5′-GTCTGGATTCTACCTGCTAC -3′ | 5′-AAAGACTTCTTGTGTGTCTGTC -3′ |
